# To Be Authentic, to Be Eco: Exploring the Link Between Authenticity and Pro-environmental Behavior

**DOI:** 10.3389/fpsyg.2021.755860

**Published:** 2021-11-12

**Authors:** Ying Yang, Shuhua Zhu, Yulu Gan, Junhua Dang

**Affiliations:** ^1^Mental Health Education and Counseling Centre, Zhejiang Ocean University, Zhoushan, China; ^2^Institute of Psychology, Chinese Academy of Sciences (CAS), Beijing, China; ^3^Department of Psychology, University of Chinese Academy of Sciences, Beijing, China; ^4^Department of Development and Planning, Zhejiang Ocean University, Zhoushan, China; ^5^Mental Health Education Centre, Zhejiang International Maritime College, Zhoushan, China; ^6^College of Education, Wenzhou University, Wenzhou, China; ^7^Department of Neuroscience, Uppsala University, Uppsala, Sweden

**Keywords:** authenticity, authentic self, pro-environmental attitude, pro-environmental behavior, personality

## Abstract

Authentic self is believed to be morally good. The current research proposes that the authentic self is also environmentally good. Across two studies, we tested the link between authenticity and pro-environmental attitude and behavior. In Study 1 (*N*=2,646), dispositional authenticity was found to be a predictor of pro-environmental behavior (PEB). In Study 2 (*N*=474), participants in the authentic condition (recalling their experiences of being authentic) were more willing to donate money to protect the environment than those in the inauthentic (recalling their experiences of being inauthentic) or the neutral (recalling their experiences of a typical day) conditions. Participants in the authentic condition also reported higher intention to conduct PEB than their peers in the other conditions. The results of the present research provide initial evidence that people are more likely to endorse pro-environmental attitude and behave pro-environmentally when being authentic.

## Introduction

Human beings are facing unprecedented environmental problems due to their own behaviors. More and more psychologists have begun to investigate this issue (Oskamp, 2000; van der Linden et al., 2015; Pearson et al., 2016). There are many possible factors influencing people’s pro-environmental behavior (PEB), such as environmental value (Schultz et al., 2005; Byrka et al., 2010), nature experiences (e.g., Rosa and Collado, 2019), and personalities (e.g., Markowitz et al., 2012; Soutter et al., 2020). The current research aims to investigate whether authenticity, a neglected yet important factor, can predict PEB and the intention to conduct PEB.

### Personality and PEB

Human’s behavior is one of the most important contributors of environmental problems (Gifford and Nilsson, 2014; Cook et al., 2016; Schmidt et al., 2017; Lange and Dewitte, 2019). Therefore, extent efforts have been devoted to examine factors that can make people behave pro-environmentally to mitigate or exacerbate environmental problems. A great deal of research has explored social and psychological influences on PEB, which refers to concrete actions behaved by the actor, (or not) deliberately, that are beneficial for the wellness of the natural environment (e.g., recycling, purchasing environment-friendly goods, and saving water or energy; Stern, 2000; Lange and Dewitte, 2019; Soutter et al., 2020). For instance, pro-environmental attitude, personal values, moral emotions, adherence to social and moral norms, personality traits were all linked with PEB (for reviews, see: Bamberg and Moser, 2007; Steg and Vlek, 2009; Gifford and Nilsson, 2014; Soutter et al., 2020). These factors are listed as examples that could influence PEB because they are more relevant to our present work than others.

Individuals’ traits or personalities, which reflect their characteristic patterns of thoughts, feelings, and behaviors, may be significant antecedents of their pro-environmental attitudes and behaviors (Soutter et al., 2020). Theorists claim that the knowledge of which personalities are related to PEB helps us develop more specific interventions for different people with particular personality traits (e.g., Soutter et al., 2020). An increasing amount of research has been conducted to explore the relationship between personality traits and pro-environmental attitudes and behaviors (Hirsh, 2010; Markowitz et al., 2012; Milfont and Sibley, 2012; Klein et al., 2019). The Big Five model of personality (Goldberg, 1990), including five personality facets (i.e., emotional stability/neuroticism, extraversion, openness, agreeableness, and conscientiousness), and the six-factor HEXACO model (which adds honesty-humility to the Big Five; Ashton and Lee, 2007), are the most often used personality models.

Among the five or six personality domains, only the relationship between openness and pro-environmental attitudes and behaviors has been consistently found, whereas the findings regarding the association between other personality facets and pro-environmental attitudes and behaviors are inconsistent (Hirsh and Dolderman, 2007; Markowitz et al., 2012; Gifford and Nilsson, 2014; Brick and Lewis, 2016; Soutter et al., 2020). Such mixed results suggest that general personality traits may be too broad for PEB, which calls for more fine-grained exploration (Passafaro et al., 2015). Additionally, previous studies mainly relied on correlational design, limiting our understanding of the causal effect of personality on PEB.

In the present work, we focus on a traditionally valued but newly emphasized personality trait, authenticity, to cope with the environmental concerns. The relevant research with authenticity and the rationale of our work will be elaborated before presenting our empirical work.

### Authenticity and PEB

Both scholars and laymen cherish the value of being authentic. Many popular sayings stress the importance of being one’s authentic or true self, e.g., “know thyself,” “To thine own self be true,” “Just be your true self.” In psychology, self-authenticity has been catching more attention recently (Hicks et al., 2019; Newman, 2019; Ryan and Ryan, 2019). Our main concern here is the self-authenticity and its link with and effect on PEB.

Authenticity is defined as “*the unobstructed operation of one’s true self in one’s daily enterprise*” (Goldman and Kernis, 2002). According to the classic framework of Kernis and Goldman (2006), there are four components of authenticity, i.e., *awareness*, *unbiased processing*, *authentic behavior*, and *relational orientation*. That is, authentic people can (a) be aware of their inner motives, feelings, desires, strengths, and weaknesses; (b) unbiasedly process their self-facets; (c) act in line with their values, preferences, and needs; and (d) develop genuine, open, and trustful relationship with close others. Kernis and Goldman (2006) also developed an inventory with four subscales reflecting the four aforementioned dimensions (i.e., Authenticity Inventory). Recently, scholars found that authenticity could also be a state construct, and most people can experience state authenticity no matter their trait authenticity is high or low (Lenton et al., 2013, 2016; Slabu et al., 2014; Sedikides et al., 2017). Therefore, there is a consensus that authenticity can be either a trait or a state (Sedikides et al., 2017).

A great deal of relevant research has indicated that self-reported authenticity is linked with interpersonal and intrapersonal well-being (Wickham et al., 2016; Ryan and Ryan, 2019; Zhang et al., 2019; Sutton, 2020). Similarly, experimental studies also showed that people whose authenticity were temporarily elicited *via* a brief manipulation reported higher scores on well-being scales, regardless of their trait authenticity (Schlegel et al., 2009; Kifer et al., 2013). Furthermore, elicited authenticity can also act as a buffer against threat (e. g., rejection, negative work feedback; Vess et al., 2014; Gino and Kouchaki, 2020), and elevate the sense of meaning (Schlegel et al., 2009) and interpersonal functioning (Plasencia et al., 2016).

Relevant to our present work, researchers found that authenticity was related to morality (Gino et al., 2015). Morality is often seen as one of the characteristics of one’s authentic self (Newman et al., 2015; Ryan and Ryan, 2019; Stanley and De Brigard, 2019; Zhang et al., 2019). Even the authentic self of the enemy is deemed as moral (De Freitas and Cikara, 2018). Investigations showed that trait authenticity was negatively associated with aggressive behavior, and positively with ethical behavior (Pinto et al., 2012; McCormick et al., 2015; Knoll et al., 2016). Likewise, experimental research showed that the sense of authenticity could promote individuals’ moral behavior in diverse settings (Kim et al., 2018; Zhang et al., 2019). For instance, when participants were asked to describe the experience of being authentic to themselves, they reported higher sense of moral self-regard (Gino et al., 2015). Further, when people were asked to follow their authentic selves, they made more moral decisions and were less likely to violate moral norms (Kim et al., 2018), and were reluctant to conduct unethical behavior in working settings (Zhang et al., 2019).

Given that authenticity increases morality, we propose that authenticity can positively predict PEB and the intention to conduct PEB, because PEB also has moral implications (e.g., Schultz, 2001; Haidt and Kesebir, 2010; Markowitz and Shariff, 2012; Xu et al., 2021; for a review, see Nolan and Scultz, 2015). For example, environment protection has been assumed by researchers to be an exemplary value of the universalism, which is one of the 10 basic human values (Schwartz, 1994). Other works also showed that some personal values, e.g., biospheric (valuing the welfare of natural environment) and altruistic (valuing the wellness of other people) ones, are relevant in predicting PEB (Stern and Dietz, 1994; Bouman et al., 2018). Among lay people, PEB is positively correlated with prosocial behavior (e.g., Neaman et al., 2018; Xu et al., 2021), and can be perceived as a signal of the actor’s moral characteristics (Vesely et al., 2020). Meanwhile, individuals who endorse self-transcendent values (e.g., environmentalism) are more likely to feel moral obligations toward the environment (Karp, 1996; Schultz and Zelezny, 1998; Feinberg and Willer, 2013). Moral emotions (e.g., awe, guilt, and pride) are also found to be predictors of pro-environmental attitude and behavior (Thøgersen, 2006; Bamberg and Moser, 2007; Zhao et al., 2018; Liang et al., 2019) and enhance people’s support for costly pro-environmental policies (Lu and Schuldt, 2015, 2016).

Although, no previous work has examined such a proposition, preliminary evidence indicates that authenticity may be a predictor of PEB. As a personality trait, authenticity has overlap with the honesty-humility factor of personality (Maltby et al., 2012). Honesty-humility refers to the tendency of cooperating and not exploiting others as well as the natural environment (Ashton and Lee, 2007; Lee et al., 2015), which has been proved to be a personality correlate of PEB (for a meta-analysis, see Soutter et al., 2020).

Taken together, authenticity is likely to be a precursor of PEB. In the present work, we aim to test both the link between trait authenticity and PEB and the causal effect of state authenticity on PEB, because authenticity can be either a trait or a state (Sedikides et al., 2017). Specifically, we argue that trait authenticity is a positive predictor of PEB, and the triggered sense of state authenticity enhances PEB (intention). Our work may contribute in three ways. First, we propose a novel personality trait which may be more relevant for PEB. Second, both correlational and experimental research design are employed to examine the link between authenticity and PEB. Third, our work implicitly suppose that everyone may conduct PEB when they feel authentic, which sheds light on new interventions or policies on facilitating PEB.

### The Current Research

Two studies were conducted to test whether authenticity can predict and promote PEB. In Study 1, we tested whether individuals with high trait authenticity were more likely to behave pro-environmentally. A sample of college students were surveyed using instruments measuring authenticity and PEB. We expected that trait authenticity would be a significant predictor of PEB. In Study 2, we tested the causal effect of state authenticity on PEB by having participants recall their experiences of being authentic (vs. inauthentic and neutral) and then respond on pro-environmental intention measures and report the amount of time and money they could donate to a pro-environment activity. We expected that participants in authentic (vs. control) condition would report higher PEB intentions in the future and donate more time and money to protect environment.

## Study 1

### Method

#### Participants and Procedure

The present sample consisted of 2,646 undergraduates (1,221 females; *M*_age_=18.69, *SD*_age_=1.04, age range: 16–28) from a university in southeast China. They completed the questionnaires of the current study as a part of their mental health assessment. Participants were invited to the psychological testing room and were told to take part in freshmen mental health assessment on a computer. The questionnaires used in the current study and several demographic variables of interest (i.e., gender, age, and socioeconomic status) were included in the mental health assessment.

#### Measures

##### Authenticity Inventory

The 45-item scale was developed by Kernis and Goldman (2006) to capture the four components of authenticity: awareness, unbiased processing, behavior in accord with one’s values, and relational orientation. Sample items are: “For better or for worse I am aware of who I truly am (Awareness),” “I am very uncomfortable objectively considering my limitations and shortcomings (Unbiased Processing, reverse scoring),” “I am willing to change myself for others if the reward is desirable enough (Behavior, reverse scoring),” and “If asked, people I am close to can accurately describe what kind of person I am (Relational Orientation).” Participants rated their agreement with each statement on a seven-point scale (1=strongly disagree, 7=strongly agree). Their ratings were summed and averaged, with higher score indicating higher trait authenticity (Cronbach’s *α*=0.89; *M*=4.60, *SD*=0.57).

##### Pro-environmental Behavior

Participants were asked to report their behavior frequencies of conducting each of 12 kinds of PEB adapted from previous work (Schultz et al., 2005). These items include a variety of daily activities related to eco-friendly lifestyle such as “looked for ways to reuse things,” “recycled cans or bottles,” “conserved gasoline by walking, cycling, or taking public transportation.” Items were rated on a five-point scale from 1 (never) to 5 (very often). The scores were summed and aggregated to generate an index of PEB in daily life (*α*=0.82; *M*=3.05, *SD*=0.58).

### Results

To test our predictions, we first conducted correlational analyses between authenticity and its four dimensions and PEB. Then regression analysis was performed, in which authenticity total score and PEB were set as independent and dependent variables, respectively, while gender, age, and socioeconomic status were taken as control variables.

As predicted, the correlation between dispositional authenticity and PEB frequencies was significant, *r*=0.35, *p*<0.001 (see Table 1). The four dimensions of dispositional authenticity were all significantly correlated with PEB, *r*s>0.19, *p*s<0.001. Regression analysis showed that dispositional authenticity was a significant predictor of PEB, even after controlling for gender, age, and socioeconomic status, *β*=0.35, *R*^2^=0.12, *F* (4, 2,641)=92.35, *p*<0.001. The results provided strong evidence for our proposal that individuals with higher dispositional authenticity are more likely to behave pro-environmentally.

**Table 1:** Descriptive and correlational statistics in Study 1.

*N*=2,646	*α*	*M* (*SD*)	1	1.1	1.2	1.3	1.4
1. Authenticity	0.89	4.60 (0.57)	--				
1.1 Awareness	0.85	4.80 (0.83)	0.84^***^	--			
1.2 UP	0.77	4.47 (0.84)	0.76^***^	0.48^***^	--		
1.3 Behavior	0.44	4.18 (0.55)	0.73^***^	0.52^***^	0.39^***^	--	
1.4 RO	0.75	4.94 (0.70)	0.76^***^	0.54^***^	0.36^***^	0.46^***^	--
2. PEB	0.82	3.05 (0.58)	0.35^***^	0.33^***^	0.19^***^	0.25^***^	0.32^***^

Awareness and Behavior, as well as UP and RO are four dimensions of Authenticity Inventory. UP, unbiased process; RO, relational orientation; and PEB, pro-environmental behavior.

*** *p*<0.001.

## Study 2

In Study 2, we manipulated participants’ state authenticity by having them recall authentic experience (vs. inauthentic and neutral experience). After the manipulation, participants were asked to indicate their intention to implement PEB in the future. We expected that participants in authenticity condition would like to behave more pro-environmentally than their counterparts in the other two conditions.

### Method

#### Participants

Participants were 474 college students (310 females, *M*_age_=18.97, *SD*_age_=0.87) from a university located in southeast China. They received course credits for compensation.

#### Procedures and Materials

Participants were firstly asked to give their consent to participate the study and then randomly assigned to one of the three conditions: authenticity, inauthenticity, and neutral. The manipulation procedures were adapted from previous research, which has been demonstrated to be reliable and valid (Kifer et al., 2013). Participants were instructed to recall and write an experience in which they felt authentic or inauthentic or neutral. In the (in)authenticity condition, the instructions went as follows:

*Please take a few minutes to recall a particular event in which you felt authentic [inauthentic]. To be authentic [inauthentic] means that you are (not) true to yourself and behaved in accordance with your true thoughts, beliefs, personality, and values. Try to re-experiecne the situation and describe what happened and where it happened, how you felt, etc. Please write down at least five lines*.

In the neutral condition, participants were asked to recall what they did during a typical day and write down at least five lines.

Immediately after the recalling task, participants were asked to answer manipulation check questions on a seven-point scale (1=strongly disagree, 7=strongly agree). The question, “I felt my true self just now,” was used to test if the recalling task induced the experience of authenticity (*M*=5.13, *SD*=1.57).

Next, participants responded to two measures of the intention to conduct PEB as two ostensibly different tasks. One measure was adapted from the 12-item scale used in Study 1, to examine participants’ intention to behave pro-environmentally in their daily lives in the future. Sample items were: “I will look for ways to reuse things in my daily life” and “I will recycle cans and bottles in my daily life.” In the present study, we used a seven point likert scale (1 = strongly disagree, 7 = strongly agree). The scores of the 12 items were summed and aggregated to generate an index of PEB intention in future’s life (*α*=0.87; *M*=5.26, *SD*=0.85).

To further assess participants’ PEB intention, we developed a scenario in which an environment protection organization named *Paradise Foundation* was recruiting volunteers to take part in pro-environmental activities (e.g., picking up garbage, cleaning the beach) and soliciting money to protect natural resources. Participants were asked to indicate (1) whether they were willing to engage in, and how many hours per month they could spend on it (0=will not participate, 1–16h per month=the range of the time they could choose, *M*=2.77, *SD*=3.70), and (2) how much money they would like to donate for protecting the environment (0=will not donate, RMB 1–100=the range of the money they could choose, *M*=15.22, *SD*=25.90). Participants were also asked to leave their phone number and were told that they would be contacted *via* the phone number they left and asked to spend time and/or donate money according to the amount they wrote in the survey.

Finally, participants were thoroughly debriefed and thanked. Specially, they were told that the environment protection organization was fictitious, and they would not actually need to spend time and/or donate money. No participant was suspicious about the true aims of the study and all participants’ data was analyzed.

### Results

#### Manipulation Check

To test whether the manipulation procedures successfully elicited authenticity or inauthenticity, one-way ANOVA with *post hoc* analyses were conducted. As predicted, the manipulation was valid. One-way ANOVA with the manipulation check item as the dependent measure showed the effect of the manipulation was significant, *F* (2, 471)=27.13, *p*<0.001, *η*^2^=0.10, 90% CI=[0.07, 0.15]. *Post hoc* analysis showed that participants in the authenticity condition (*M*=5.72, *SD*=1.20) reported higher sense of being the true self than those in the inauthenticity condition (*M*=4.48, *SD*=1.76), *p*<0.001, and those in the neutral condition (*M*=5.18, *SD*=1.44), *p*=0.001. Participants’ sense of being the true self in the inauthentic condition was significantly lower than that in the neutral condition, *p*<0.001.

#### Hypothesis Testing

Again, a series of one-way ANOVA with *post hoc* analyses were conducted to test whether our outcome variables (i.e., PEB intention, amount of money and time donated to protect environment) varied across experimental conditions.

First, regarding the first measure of PEB intention (i.e., intention to behave pro-environmentally in future daily life), one-way ANOVA showed the effect of the manipulation was significant, *F* (2, 471)=3.59, *p*=0.028, *η*^2^=0.02, 90% CI=[0.00, 0.04]. Participants in the authenticity condition (*M*=5.41, *SD*=0.80) reported more willingness to act pro-environmentally in future daily life, compared to their counterparts in both the inauthentic condition (*M*=5.16, *SD*=0.86, *p*=0.010), and the neutral condition (*M*=5.22, *SD*=0.88, *p*=0.052). The difference between the inauthentic and the neutral condition were not significant, *p*=0.504.

Second, the effect of the manipulation on the money participant intended to donate for protecting the environment was significant, *F* (2, 471)=3.38, *p*=0.035, *η*^2^=0.01, 90% CI=[0.00, 0.04]. Participants in the authentic condition (*M*=19.63, *SD*=29.97) would like to donate more money to protect the environment than their counterparts in both the inauthentic (*M*=13.34, *SD*=24.19, *p*=0.032) and the neutral condition (*M*=12.84, *SD*=22.76, *p*=0.019). The difference between the inauthenticity and the neutral condition was not significant, *p*=0.862. However, there was no significant difference among these three conditions regarding the time participants would like to spend on pro-environmental activities, *F* (2, 471)=1.38, *p* = 0.253, *η*^2^=0.01, 90% CI=[0.00, 0.02].

## General Discussion

The present work, to our best knowledge, is the first to examine the link between authenticity and PEB. Across two studies, we tested the correlational and causal link between authenticity and PEB (intention). As predicted, dispositional authenticity was found to be a significant predictor of PEB (Study 1). The relationship between authenticity and PEB in the present work (*r*=0.35) was stronger than other personality facets showed in previous work (*r*=0.09–0.25; for a meta-analysis, see: Soutter et al., 2020), suggesting that authenticity may be a stronger personality precursor of PEB relative to other most studied personality traits. Furthermore, the induced feeling of authenticity also increased participants’ willingness to behave more pro-environmentally than the inauthentic and the neutral feelings (Study 2), providing causal evidence for the effect of authenticity on PEB. These findings suggest that the authentic self is also environmentally good, extending the notion that the authentic self is morally good (Strohminger et al., 2017).

### Implications

Replicating and extending previous work (Markowitz et al., 2012; Soutter et al., 2020), our work showed that individual differences and/or personalities are factors affecting PEB. Trait authenticity has overlap with the dimension of honesty-humility personality (Maltby et al., 2012), which is proved to be a significant predictor of PEB (Soutter et al., 2020). The current work provided direct evidence for the proposed link between authenticity and PEB. Moreover, the present findings also shed light on the causal link between personality (i.e., authenticity) and PEB by manipulating state authenticity. Overall, our work suggests that authenticity can encourage people to behave pro-environmentally.

There are several theoretical and practical implications of the current work. Theoretically, the findings may help to advance the understanding of precursors of PEB, as well as the moral feature of authenticity. First, authenticity, as a personality trait, was demonstrated in the present work to be an important predictor of PEB. Although much work has been done to reveal the predictive role of personality in predicting PEB, less consistent conclusions have been obtained (Hirsh and Dolderman, 2007; Markowitz et al., 2012; Gifford and Nilsson, 2014; Brick and Lewis, 2016; Soutter et al., 2020). Some scholars suggested that the personality model often used in previous work may not be relevant for PEB, because the Big Five or Six are general personalities that may be too broad when predicting PEB (Passafaro et al., 2015). Instead, the morality implication of authenticity makes it a narrower trait that is more predictive for PEB. Therefore, the current work helps to deepen the understanding of the relationship between personality and PEB, especially the pro-environmental effect of authenticity.

Second, the present work also deepened the understanding of who (under what conditions) will conduct PEB. Almost all the scholars investigating personality and PEB implicitly suppose that some people with certain personalities are less likely to behave pro-environmentally. Our work, however, suggests that everyone seems to be willing to conduct PEB. Specifically, Study 2 randomly assigned participants to recall authentic or control (inauthentic or neutral) experiences. The results showed that participants in the authentic condition reported higher pro-environmentality than those in control conditions, regardless of their trait authenticity level.

Third, the present findings indicate that environmental friendliness is one of the features of authentic self, replicating and extending previous work on the morality effect of authenticity. Authentic self is moral, which is supported by much previous work (for a review, see Strohminger et al., 2017). We demonstrated that authenticity also has pro-environmental implications. Because being authentic increases morality and PEB is considered as morally good, being authentic can increase PEB. Similarly, authenticity may also promote other behaviors that are considered as morally good, which could be empirically tested by future studies.

Practically, the current results suggest that individuals with high authenticity are willing to protect the only planet for human beings, which brings good news for both theorists who study sustainability and policymakers who advocate sustainability. First, policymakers should not regard people who hurt the environment as totally evil, because these people may also want to protect the environment when their sense of authenticity is triggered. Therefore, besides the punishment measures, some encouragement measures, which can stimulate people’s intrinsic motivation to protect the environment in daily life, may be more effective. Second, the current findings may be taken into consideration to develop PEB interventions or frame PEB advertisement or information. The measures or information should be authentic or enable people to behave authentically. For example, PEB interventions should make targets behave autonomously or have the free choice, which are essential elements of authenticity (Ryan and Ryan, 2019). The information regarding the PEB advertisement should also be authentic, which is more compelling, as suggested by a recent investigation, which showed that advertisement authenticity was a significant predictor of customers’ pro-environmental behavior (Kaur et al., 2021). Third, it is especially important to induce the public’s authenticity when advocating to protect the environment. More directly, we can develop strategies to convince the public to believe that their authentic selves are pro-environmental. For example, we may remind the public that everyone has conducted PEB, which has been proved to be a motivator of acting pro-environmentally (Geng et al., 2016).

### Limitations and Future Directions

Although, the present findings deepen the understanding of the factors influencing PEB from the authentic-self perspective, there are several limitations. First, in Study 2, the outcome variable of the causal association between authenticity and PEB was behavioral intention, rather than actual PEB. Even though, we measured people’s actual PEB frequencies in their everyday lives in Study 1, there could be memory biases. Therefore, it is needed to test the positive effect of authenticity on sustainability using measures of actual PEB with higher ecological validity. Second, all participants in our studies were Chinese college students. Future research is needed to replicate the study in other cultures with more diverse samples. Third, the effect size of Study 2 is not large, although, the effect is statistically significant. Replications of the present work are required to confirm the effect of authenticity on PEB.

## Conclusion

The authentic self is pro-environmental. The present work provides a new perspective to study individuals’ daily behavior for sustainability. Authenticity is likely to be a personality trait more relevant for PEB, although, further work is needed to replicate and extend it. The current findings showed that the sense of authenticity may help one be aware of the ecological crisis and more likely to take actions to contribute to it (e.g., acting pro-environmentally in daily life and donating money to preserve the resources). Future work can replicate the findings in diverse cultural contexts and may also develop theoretical framework regarding authenticity and PEB. Furthermore, the role of authenticity in predicting and promoting PEB can be taken into consideration in pro-environmental practices.

## Data Availability Statement

The original contributions presented in the study are publicly available. This data can be found here: https://osf.io/jbwa3.

## Ethics Statement

The studies involving human participants were reviewed and approved by Zhejiang Ocean University. The patients/participants provided their written informed consent to participate in this study.

## Author Contributions

YY, SZ, and JD developed the initial research idea and designed the research. YY, SZ, and YG conducted and analyzed the studies and collected the data. All authors wrote and revised the manuscript. All authors contributed to the article and approved the submitted version.

## Funding

The research is supported by the Department of Education of Zhejiang Province [2018QN001].

## Conflict of Interest

The authors declare that the research was conducted in the absence of any commercial or financial relationships that could be construed as a potential conflict of interest.

## Publisher’s Note

All claims expressed in this article are solely those of the authors and do not necessarily represent those of their affiliated organizations, or those of the publisher, the editors and the reviewers. Any product that may be evaluated in this article, or claim that may be made by its manufacturer, is not guaranteed or endorsed by the publisher.
